# Supplementary figures and images for: Analogs of 6-Bromohypaphorine with Increased Agonist Potency for α7 Nicotinic Receptor as Anti-Inflammatory Analgesic Agents
Source: Mar Drugs. 2023 Jun 20;21(6):368. doi: 10.3390/md21060368 (PMC10305119; doi:10.3390/md21060368)

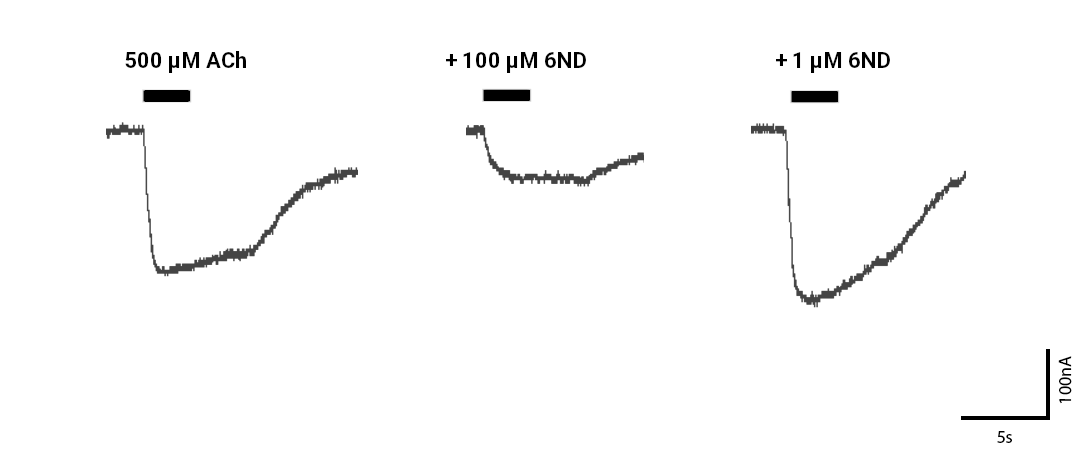

Supplement: Supplementary file 1 [file marinedrugs-21-00368-s001.zip › Figure S1.png]

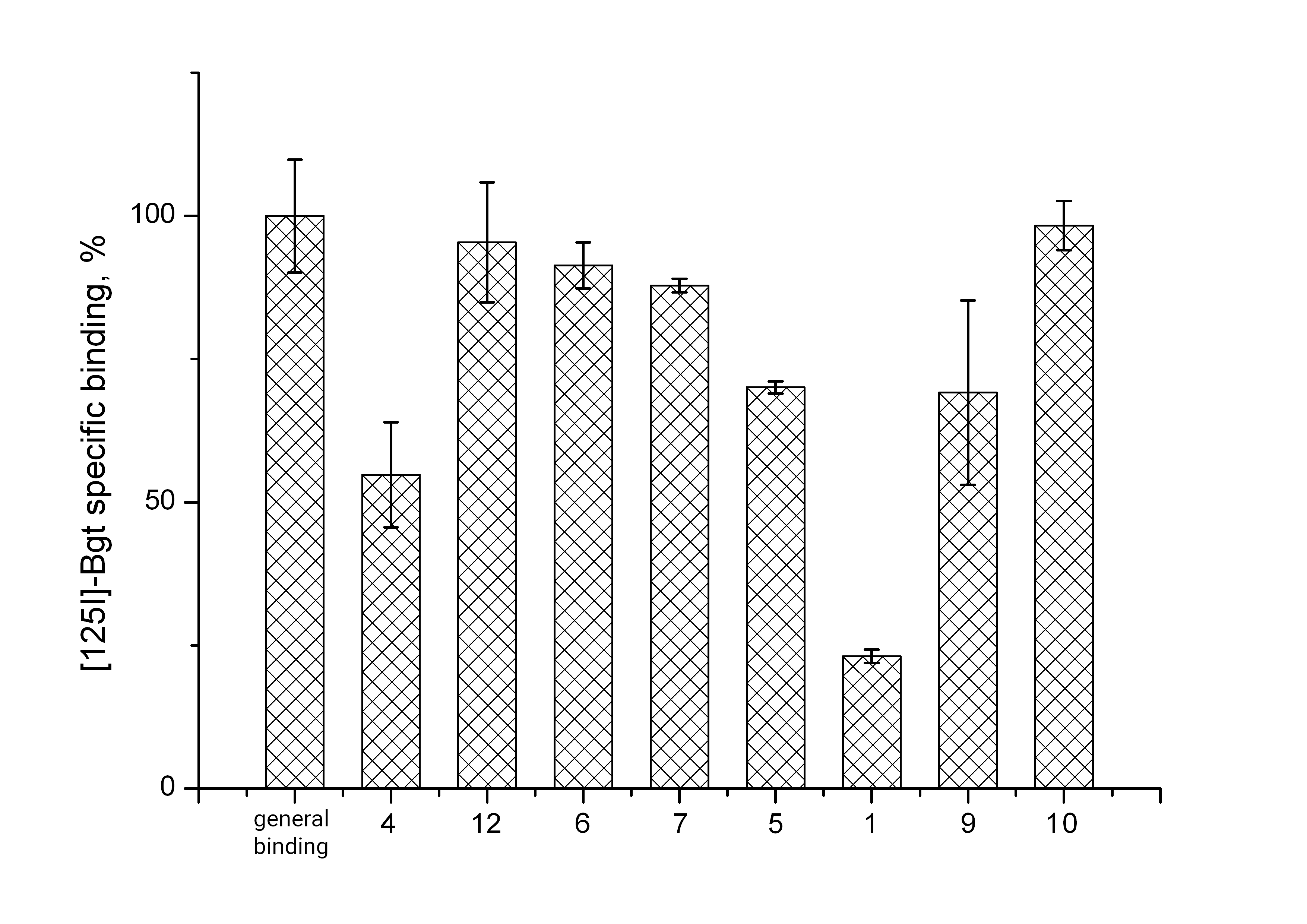

Supplement: Supplementary file 1 [file marinedrugs-21-00368-s001.zip › Figure S2.png]
